# Literary Notes

**Published:** 1903-06

**Authors:** 


### LITERARY NOTES.

THE Maltine Company has issued recently an album of photographs, called Quarantine Sketches. It contains between thirty and forty engravings showing the care of imigrants at the New York quarantine station on Ellis Island, in all its phases from the arrival on shipboard to the departure by rail for the far west. It is altogether one of the most instructive booklets that has been published by the Maltine Company; and this is according it great praise, for the company is noted for the dissemination of useful material.

---

THE Antikamnia Chemical Company has published a reference chart of Diseases of the Nervous System and Muscles, which is being distributed to the medical profession. It is prepared by Professor Edward Curtis Hill, M.D., of the Denver and Gross Medical College, Denver, Colorado, and is issued in folder form, making it convenient for the pocket. A well prepared index adds to its utility.

---

THE Samuel D. Gross prize of $1,200 will be awarded January 1, 1905, for the best original essay not exceeding 150 octavo pages in length, illustrative of some subject in surgical pathology or surgical practice, the candidates for the prize to be American citizens. For particulars address the committee, Drs. John B.

Roberts, William L. Rodman, and William J. Taylor, Philadelphia.

THE *International Medical Magazine* for June, 1903, will be devoted to a symposium on Hyperchlorhydia. Among the contributors the following names are announced: Professors C. A. Ewald and Rosenheim, of Berlin; George Hayem, of Paris; Carl von Noorden, of Frankfurt, and Dr. L. Kuttner, of Berlin. Also Drs. John C. Hemmeter and Boardman Reed, of Philadelphia; Allen A. Jones, of Buffalo; John A. Lichty, of Pittsburg; Fenton B. Turck, of Chicago; A. Robin, of Newark, Delaware, and Max Einhorn, of New York.

MESSRS. LEA BROTHERS & CO. announce that conforming to many requests they issue the work on the Eye, Nose, Throat and Ear, edited by Drs. Posey and Wright, in two volumes, as well as in a single volume.

Volume I., will be known as Posey on the Eye, and covers completely the subject of ophthalmology. It contains 690 pages, 358 engravings and 19 plates in colors, and monochrome. Price, cloth, $4.00 net.

Volume II. will be known as Wright on the Nose, Throat and Ear, and contains 570 pages, 292 engravings and 16 colored plates. Price, cloth, $3.50, net. The convenience of this plan, especially for textbook puropses, is obvious.

The work will continue also to be published in a single volume. Price, cloth, $7.00; leather, $8.00, net.

*The Medical Critic*, of New York, has issued an *index medicus* for 1902, which is a standard octavo book in paper binding of 268 pages. It begins with a list of the medical journals of the world, then follows a catalogue of the medical books of the year, next a classified list of magazine titles, which constitutes the bulk of the volume, and, finally, a roster of physicians who died during the year. It is a work of surprising accuracy, represents an enormous amount of labor, a considerable money outlay, and a spirit of progressiveness that is to be highly commended. It deserves the united moral and financial support of a grateful profession.

